# Treatment of Eczema

**Published:** 1885-10

**Authors:** T. C. Osborn

**Affiliations:** Cleburn, Texas, Hon. Mem. T. S. M. A., Etc.


### CORRESPONDENCE.

### The Treatment of Eczema.

*Letter from Dr. T. C. Osborn, Cleburn, Texas, Hon. Mem. T. S. M. A., etc.*

Editor Daniel's Texas Medical Journal:

AS the subject is just now so freely discussed in all the medical journals of the United States, and as the disease is more numerous than all other affections of the skin combined, and especially as there is so great a diversity in regard to its proper treatment, I feel it my privilege, as well as my duty, to add to its literature the means, which in my hands, has been, on all occasions and under every variety of the disease, as successful as could be wished for. I mean

ECZEMA.

Billard says "the treatment of chronic eczema is as difficult in its choice and direction as it is uncertain in its results;" but he so wrote in 1840, since which time the nature of the eruption has become much better understood, and its principles of treatment more effectual in arresting its progress.

Devergie states that it "constitutes one-third of his cases," and later dermatologists not only confirm this fact, but go further, and tabulate it as more frequent than all the other eruptive diseases together.

Under these circumstances, anything that promises speedy and perfect relief from eczema cannot be easily overestimated.

For forty years I was in the habit of using only citrine ointment as a local application, which, although very effectual in curing the disease, was exceedingly painful to children, and I was ever wishing for something less troublesome to use, but it was not until 1880 that I succeeded in finding in chaulmoogra oil an agent equally effectual, and not at all painful in its effects. Since that date I have had ample opportunities of testing its virtues in all the forms of eczema, and am so well satisfied with its results that there has been no reason for my changing it for any other remedy.

The formula for the administration of this oil is:

| | | |
|---|---|---|
| Oil Chaulmoogra } | | |
| Oil Cajeputi } | a. a. | ℥j |
| Tinct. Camphorœ | | f℥ij |

M. ft. Linimentum. Sig. Apply by rubbing with the naked hand over the eruption morning and night, until the disease is cured. A very few days suffices to cure the eruption, and the itching is arrested by the first application.

But I have by experiment found that the disease will assuredly return, unless the secretions are kept corrected by the following mixture:

| | |
|---|---|
| Magnesia Sulphas | ℥iij |
| Ferri Sulphas | ʒij |
| Aq. Cinnamomi | ℥xij |
| Acid Sulphurici diluti | ʒjvss |
| Tinct. Auranti Cort. | ℥iijss |
| Oil Menth pip. | gtts xx |

M. Sig. Tonic aperient. Dose, for adults, one tablespoonful two or three times daily.

This formula I took from Tilbury Fox, and find it so efficient that there is no necessity for my using mercurials, which seems to be coming into popular favor with the profession in the South.

I am quite satisfied that a few trials of this treatment will convince the most sceptical practitioner of its safety and competency in eradicating the disease I am now discussing, as well as its adaptability in the treatment of many other forms of skin affections.

In that form of eczema capitis where we have inveterate scabbing, it is, of course, always necessary not only to remove the crusts, but the hair also, by shaving.

Hoping I am in time for your October issue, I beg leave to subscribe myself yours cordially, T. C. Osborn, M. D.

